# Mismatch Responses Evoked by Sound Pattern Violation in the Songbird Forebrain Suggest Common Auditory Processing With Human

**DOI:** 10.3389/fphys.2022.822098

**Published:** 2022-03-03

**Authors:** Chihiro Mori, Kazuo Okanoya

**Affiliations:** ^1^Department of Life Sciences, Graduate School of Arts and Sciences, The University of Tokyo, Tokyo, Japan; ^2^RIKEN Center for Brain Science, Wako, Japan

**Keywords:** songbird, mismatch negativity, mismatch response, caudomedial nidopallium (NCM), local field potentials

## Abstract

Learning sound patterns in the natural auditory scene and detecting deviant patterns are adaptive behaviors that aid animals in predicting future events and behaving accordingly. Mismatch negativity (MMN) is a component of the event-related potential (ERP) that is reported in humans when they are exposed to unexpected or rare stimuli. MMN has been studied in several non-human animals using an oddball task by presenting deviant pure tones that were interspersed within a sequence of standard pure tones and comparing the neural responses. While accumulating evidence suggests the homology of non-human animal MMN-like responses (MMRs) and human MMN, it is still not clear whether the function and neural mechanisms of MMRs and MMN are comparable. The Java sparrow (*Lonchura oryzivora*) is a songbird that is a vocal learner, is highly social, and maintains communication with flock members using frequently repeated contact calls and song. We expect that the songbird is a potentially useful animal model that will broaden our understanding of the characterization of MMRs. Due to this, we chose this species to explore MMRs to the deviant sounds in the single sound oddball task using both pure tones and natural vocalizations. MMRs were measured in the caudomedial nidopallium (NCM), a higher-order auditory area. We recorded local field potentials under freely moving conditions. Significant differences were observed in the negative component between deviant and standard ERPs, both to pure tones and natural vocalizations in the oddball sequence. However, the subsequent experiments using the randomized standard sequence and regular pattern sequence suggest the possibility that MMR elicited in the oddball paradigm reflects the adaptation to a repeated standard sound but not the genuine deviance detection. Furthermore, we presented contact call triplet sequences and investigated MMR in the NCM in response to sound sequence order. We found a significant negative shift in response to a difference in sequence pattern. This demonstrates MMR elicited by violation of the pattern of the triplet sequence and the ability to extract sound sequence information in the songbird auditory forebrain. Our study sheds light on the electrophysiological properties of auditory sensory memory processing, expanding the scope of characterization of MMN-like responses beyond simple deviance detection, and provides a comparative perspective on syntax processing in human.

## Introduction

Mismatch negativity (MMN) is a component of the scalp-recorded event-related potential (ERP) that occurs in human when they are exposed to unexpected or rare sensory stimuli (Näätänen et al., 1978). Auditory MMN is typically measured by the presentation of an oddball task in which infrequent, deviant sounds are embedded in a sequence of frequent, standard sounds. Typically, the deviant sound has one acoustic feature (such as pitch or duration) that differs from the standard sound. Several studies on human MMN suggest that the main sources of MMN are located in the auditory cortex, with some contribution from the temporal, frontal, and parietal regions (Scherg and Picton, 1988; Csépe et al., 1992; Alho, 1995; Molholm et al., 2005). MMN can be elicited without attention through an automatic, pre-attentive process in the auditory cortex (Näätänen et al., 2001). This pre-attentional process is considered a useful tool to investigate the cognitive function related to recognition categories, abstract patterns, sound localization, and psychiatric and developmental disorders (Näätänen, 1995, 2003; Lopez et al., 2003; Nagai et al., 2013).

Several animals, including non-human primates (Javitt et al., 1992; Komatsu et al., 2015), dogs (Howell et al., 2011), cats (Csépe et al., 1987), rodents (Ruusuvirta et al., 1998; Umbricht et al., 2005; Ehrlichman et al., 2008; Shiramatsu et al., 2013; Christianson et al., 2014; Harms et al., 2014), and pigeons (Schall et al., 2015), show MMN-like responses, called mismatch responses (MMRs). Previous studies demonstrated that MMRs are generated from the higher-order areas of the auditory cortex in cats (Pincze et al., 2001) and rats (Shiramatsu et al., 2013). While accumulating evidence suggests the homology of non-human animal MMR and human MMN, it is still not clear whether the function and neural mechanisms of MMR and MMN are comparable. There is controversy as to whether MMR reflects sensory memory-based responses, such as MMN, or neural adaptation, such as stimulus-specific adaptation (SSA) (May and Tiitinen, 2010). Repeated exposure to the standard sound results in attenuation of neural responses in the auditory cortex, whereas rare deviant sounds elicit larger neural responses. To resolve this controversy, a number of studies have applied several control paradigms and violations of abstract rules in an attempt to elicit and record responses reflecting genuine deviance detection (Schröger and Wolff, 1996; Ruusuvirta et al., 2007; Wild et al., 2009; Ruhnau et al., 2012; Astikainen et al., 2014; Harms et al., 2014; Attaheri et al., 2015).

In this study, we investigated intracranial MMR from the songbird caudomedial nidopallium (NCM), a forebrain structure considered to be analogous to the higher-order auditory cortex in mammals (Bolhuis and Gahr, 2006). Songbirds communicate with each other by vocalization and develop a complex vocal pattern through vocal learning. The Java sparrow (*Lonchura oryzivora*), a species of songbird, is highly social and maintains communication with flock members using frequently repeated contact calls. They use seven types of calls (Goodwin and Woodcock, 1982) and emit similar but distinct calls in disparate situations of aggressiveness or affinity, which are composed of short syllables with narrow intervals repeated in quick succession (Baptista and Atwood, 1980; Goodwin and Woodcock, 1982; Furutani et al., 2018). In addition, male birds learn the song from their fathers and coordinate bill-click sound with song sequences, suggesting that non-vocal sounds are integrated with vocal courtship signals (Soma and Mori, 2015). It is crucial for the Java sparrow to process repeated sound signals and detect novel ones.

The NCM receives input from the primary area field L, which has inputs from the auditory thalamus (Vates et al., 1996). A substantial body of evidence suggests that the NCM is involved in the processing of complex natural vocalizations and memorizing of tutor song to imitate during vocal learning (Chew et al., 1996; Bolhuis and Gahr, 2006; Yanagihara and Yazaki-Sugiyama, 2016). It has been reported that neurons in NCM show stimulus-specific habituation to natural sounds such as conspecific and heterospecific vocalizations (Mello et al., 1995; Chew et al., 1996). In addition, neurons in the secondary auditory areas, i.e., NCM and caudomedial mesopallium (CMM), show response bias to deviant sounds in an oddball paradigm during multiunit and single-unit recordings in both anesthetized birds (Beckers and Gahr, 2012; Ono et al., 2016) and awake-restrained birds (Dong and Vicario, 2018). These studies suggest that the NCM integrates auditory information over both long- and short-time windows, and focal attention to auditory stimuli is not required for this process.

Furthermore, NCM neurons have been reported to show sensitivity to differences in sound sequence order (Lu and Vicario, 2014; Ono et al., 2016). The ability to extract rules from sound sequences is considered to be important for vocal learning. Human infants demonstrated the ability to extract grammatical rules in an artificial language task using made-up words (e.g., ABA grammar, such as “ga ti ga” and ABB grammar, such as “ga ti ti”) (Marcus et al., 1999). Recently, some animal studies measuring MMR reported that the auditory cortex of rats (Astikainen et al., 2014) and frontal cortex of macaques (Attaheri et al., 2015) respond to changes in a pattern of sound sequences. This suggests that some cognitive elements related to language learning, such as the ability to extract grammatical rules, might have originally evolved for more general behavioral skills.

In this study, we recorded local field potentials (LFPs) from the NCM of Java sparrows *via* chronically implanted electrodes with a wireless transmitter under freely moving conditions. We measured MMR in response to the deviant sounds in the single sound oddball task and in response to the sound element order in a triplet sequence oddball task. We expect that the songbird is a potentially useful animal model that will both further our understanding of the neural mechanisms of auditory sensory memory processing and provide a comparative perspective on syntax processing in human.

## Materials and Methods

### Subjects

Nine adult Java sparrows (5 male and 4 female birds) over 200 days post-hatch were used. Five birds were purchased from local suppliers, and the rest were birthed and raised in a breeding colony at the University of Tokyo. Birds were housed in our aviary under a 14:10 h light/dark cycle. Food and water were provided *ad libitum*. All animal experiments and housing conditions were approved by the Institutional Animal Care and Use Committee at the University of Tokyo.

### Experiment Design and Auditory Stimuli

Auditory stimuli were presented to the subjects from a speaker positioned at 15 cm from where the subjects sat on the perch. The sound pressure level was approximately 70 dBA when measured around the bird’s head (NL-27; Rion). Subjects remained on the perch for the majority of the time when auditory stimuli were played. Sounds were synthesized using sound analysis software (SASLab Pro; Avisoft Bioacoustics). To acclimate subjects to the experimental setup, they were placed in a plastic cage (150 mm × 305 mm × 220 mm) in the experimental chamber (600 mm × 500 mm × 500 mm) at least 30 min before the first session.

### Oddball Paradigm

**FIGURE 1:**
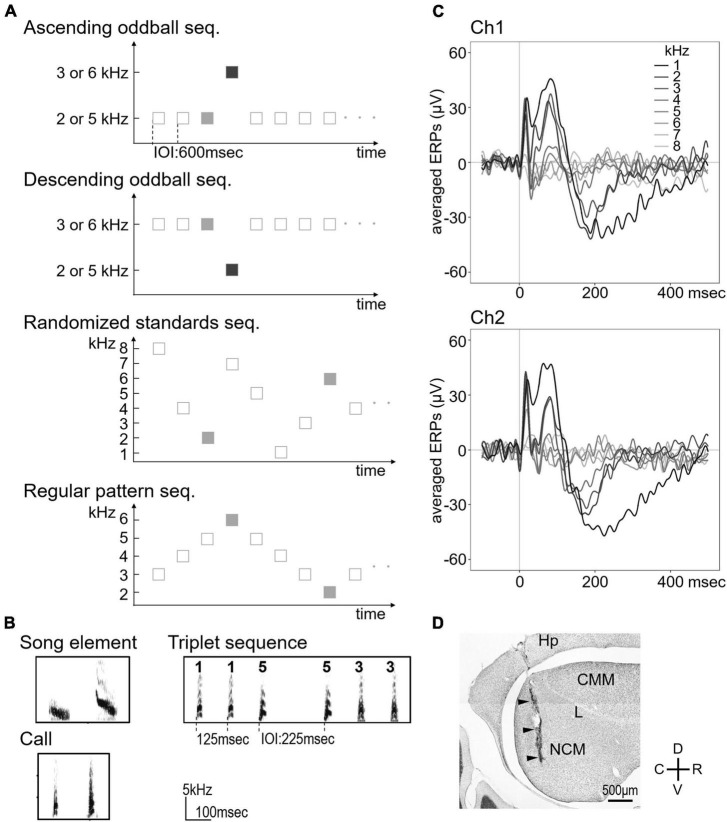
Sequence designs used in the passive auditory paradigm and averaged event-related potentials (ERPs) to pure tones. **(A)** Schematic diagram of the oddball paradigm. Deviant stimuli (dark gray squares) were presented randomly in ascending and descending oddball sequences. Light gray squares indicate standard/control stimuli to be analyzed for neural responses. **(B)** Sound spectrograms of the song elements and contact calls used as auditory stimuli (left). Examples of the triplets used in the triplet sequence (right). **(C)** Averaged ERPs were recorded from each hemisphere in response to the randomized standard sequence (*n* = 7, 1–8 kHz presented at an equal probability of *p* = 0.125). There was no significant difference in ERPs between the two channels in all experiments. **(D)** Representative Nissl-stained parasagittal section showing the location of the electrode. ERP, event-related potential; Hp, hippocampus; CMM, caudomedial mesopallium; L, field L; NCM, caudomedial nidopallium; D, dorsal; V, ventral; R, rostal; C, caudal. Arrowheads indicate the electrode tract.

Three types of sound pairs were used for ascending and descending oddball sequences (Figures 1A,B): (1) two pairs of pure 2 vs. 3 kHz and 5 vs. 6 kHz tones (duration: 50 ms, rise/fall times: 8 ms), (2) two types of song elements (duration: 75 ms, peak frequency: 2.0 and 4.3 kHz, and entropy: 0.297 and 0.339) from a bird, and (3) two calls from two individuals (duration: 26 ms, peak frequency: 2.9 and 3.7 kHz, and entropy: 0.309 and 0.484). Two sequences were presented for each sound pair with one stimulus serving as the frequent standard stimulus (87.5%) the other as the rare deviant stimulus (12.5%), and vice versa. Sound stimuli were pseudo-randomly delivered in sequences with a 600-ms inter-onset interval (IOI) between sounds. Standard and deviant stimuli presented a total of 4,400 times in each sequence using MATLAB (The MathWorks, Inc., Natick, MA, United States). In addition, two control procedures using pure tone oddball sequences were conducted to verify the influences of the probability and predictability of stimuli (Figure 1A). To investigate whether changing the probability affects the response, eight frequencies (1, 2, 3, 4, 5, 6, 7, and 8 kHz) were presented with a probability of 12.5% in a pseudorandom order as a sequence that prompted the same level of adaptation as the deviant (termed “randomized standards sequence”). To investigate the effect of predictability on the response, five frequencies (2, 3, 4, 5, and 6 kHz) were presented in a regular pattern from low to high frequency and then back down to low frequency, repetitively (termed “regular pattern sequence”). In this sequence, the highest and lowest frequency stimuli corresponded to the deviant in the oddball sequence (pure 2 kHz tones of descending oddball sequence in a pair of 2 vs. 3 kHz tones and 6 kHz tones of ascending oddball sequence in a pair of 5 vs. 6 kHz tones) and were presented with the same probability as in the oddball sequence. The second highest and lowest frequency stimulus corresponded to the standard in the oddball sequence.

### Triplet Sequence Paradigm

Triplet sound sequences (Figure 1B) were synthesized using five calls (duration: 21.2 ± 3.1 ms, peak frequency: 2.84 ± 0.48 kHz, entropy: 0.33 ± 0.03, and mean ± SD) from five Java sparrows (2 male and 3 female birds). The IOI between each call was 125 ms and between each triplet sequence was 225 ms, which was presented in groups of three based on the paradigm suggested by Astikainen et al. (2014). AAB types, which consisted of two identical calls followed by a different call, were presented as the standard sequences (16 different variants, 90%). The deviants consisted of two different types as follows: (1) “pattern-obeying deviants” that had the same AAB type pattern as the standards but differed physically (two different variants, 5%) and (2) “pattern-violating deviants” that were ABB type and thus differed from the standard sequences both physically and in pattern type (two different variants, 5%). All stimulus types were pseudo-randomly presented 3,600 times.

### Surgery and Electrophysiological Recordings

The birds were anesthetized with pentobarbital (6.48 mg/ml; 60 μl/10 g body weight) by intraperitoneal injection, and their heads were fixed in a stereotaxic apparatus with ear bars and a beak holder. Then, recording electrodes (Platinum-Iridium wires coated with Teflon with bare tips, 0.127 mm diameter, impedance 5 MΩ) of a wireless dual-channel transmitter (weight: 2.3 g, EPOCH-T2; Biopac Systems Inc., Goleta, CA, United States) were stereotactically implanted into bilateral NCM. The birds were allowed to recover for at least 3 days after surgery before the first recordings. We recorded LFP in freely behaving birds. Each channel of LFP signals was amplified 800-fold, sampled at 100 Hz (voltage range: ± 2.5 mV) with the transmitter, sent to a receiver (EPOCH-RAT-EEG-SYS; Biopac Systems Inc.), and stored on a PC using an acquisition unit (CED Power 1401) and software (Power 1401 and Spike2; Cambridge Electronic Design Ltd., Cambridge, United Kingdom). Figure 1C shows the ERPs elicited by each of the different frequencies in the randomized standard sequence. After all of the experiments, the birds were deeply anesthetized by an overdose of pentobarbital and perfused with 1 × PBS and then 4% paraformaldehyde/1 × PBS. Sagittal sections, 40 μm thick, were cut on a freezing microtome and stained with cresyl violet to verify the electrode position (Figure 1D).

### Electrophysiological Data Analysis

Local field potential data processing was performed offline using MATLAB and the open-source toolbox EEGLAB (Delorme and Makeig, 2004). LFP signals were band-pass filtered at 0.1–50 Hz and were analyzed in 600-ms epochs (100 ms before and 500 ms after stimulus onset) using baseline correction over a 100-ms pre-stimulus interval. Epochs with body movement artifacts were excluded manually. Raw recording data were averaged across all recording conditions for each channel. The mean amplitudes of ERP components were analyzed using repeated-measures ANOVAs. We treated each channel as independent recording data because electrode positions, such as anterior-posterior axes and depth, within an individual were varied in the NCM. Therefore, we investigated the difference of the data recorded from left and right hemispheres as a between-subject factor to consider laterality. For the single sound oddball paradigm, the between-subject factor was channel (left and right). The within-subject factors were stimulus type (standard and deviant) and frequency of sound (2, 3, 5, and 6 kHz), or song element type or call type. For the triplet sequence paradigm, the between-subject factor was channel (left and right). The within-subject factors were stimulus type (standard “AAB,” pattern-obeying deviants “AAB,” and pattern-violating deviants “ABB”) and time window (95–120 ms, 215–240 ms). The significance level was set at *p* < 0.05.

## Results

### Event-Related Potentials to Deviant Stimuli Negatively Shifted in the Oddball Sequence

**FIGURE 2:**
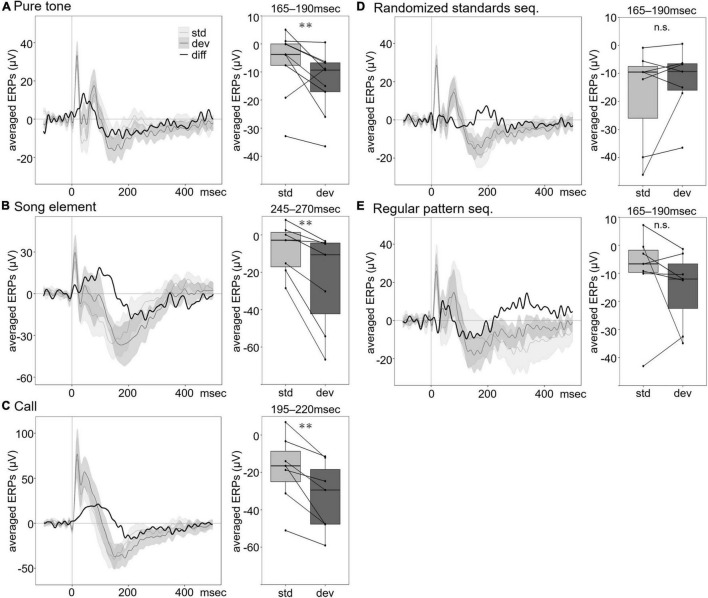
Averaged ERPs to standard and deviant stimuli and averaged ERP amplitude at the specified time window. Group averaged ERPs to the oddball standard (light gray line) and deviant (dark gray line) stimuli for pure tones **(A)**, song elements **(B)**, and calls **(C)**. Comparison between group averaged ERPs of deviant stimuli in the oddball sequence and that of the standard stimuli in the randomized standard sequence **(D)** and the regular pattern sequence **(E)**. Black line shows the difference wave obtained by subtracting the ERP to standard stimuli from the ERP to deviant stimuli. The shading indicates the range of 95% CI of the waves. In the box plot on the right of the waveform, each box shows the quartiles of the ERPs to standard (light gray) and deviant (dark gray) stimuli at each time window (pure tone: 165–190 ms, song element: 245–270 ms, and call: 195–220 ms, after stimulus onset) in each sound sequence. Each dot represents the averaged ERPs for each individual (pure tone: *n* = 9, song element: *n* = 7, and call: *n* = 7). Averaged ERPs to deviant stimuli negatively shifted, compared with that of standard stimuli in the single oddball sequence, while no significant difference was found compared with averaged ERPs to standard stimuli in the randomized standard sequence (*n* = 7) and the regular pattern sequence (*n* = 7) (repeated measures ANOVA, ^**^p < 0.01). n.s., no significance.

For the pure tone oddball paradigm, the group averaged auditory evoked potential consisted of an initial positive peak at approximately 20 ms, a negative peak at 50 ms followed by a positive peak latency of approximately 80 ms, and a broad negative component with a peak at approximately 150–200 ms (Figure 2A). A difference waveform was obtained by subtracting the standard-evoked ERP from the deviant-evoked ERP. A mean amplitude measure was extracted over a 165–190 ms latency window corresponding to the negative peaks of the difference waveform. To evaluate the differences between the ERPs, the mean amplitudes of ERP components were analyzed using repeated-measures ANOVAs. The ERPs to the deviant significantly shifted (*F*_1_,_12_ = 18.0290, *p* = 0.0011). In addition to the main effects of stimulus type, 2 and 3 kHz pure tones elicited larger amplitude ERPs than the 5 and 6 kHz tones (*F*_3_,_36_ = 8.9812, *p* = 0.0001). There was no significant difference in ERPs between the two-channel positions (*F*_1_,_12_ = 0.0606, *p* = 0.8097).

For the song element oddball paradigm, the group averaged auditory evoked potential was characterized by a positive peak at approximately 10 ms, a negative peak at 30 ms followed by a positive peak latency of approximately 45 ms, and a broad negative component with a peak at approximately 150–300 ms (Figure 2B). Difference waveforms consisted of a negative peak at 245–270 ms. We analyzed the differences between the mean ERP amplitudes extracted over a 245–270 ms latency window using repeated-measures ANOVA. Significant differences between standard and deviant ERPs were confirmed (*F*_1_,_10_ = 14.7813, *p* = 0.0032). Differences in ERPs between channels were not detected (*F*_1_,_10_ = 0.0077, *p* = 0.9317).

For the call oddball paradigm, the averaged auditory evoked potential consisted of an initial positive peak at approximately 20 ms, a second positive peak at 35 ms, followed by a third peak latency of approximately 50 ms, and a broad negative component with a peak at approximately 150–250 ms (Figure 2C). The mean ERP amplitude at 195–220 ms, corresponding to the negative peaks of the difference waveform, was analyzed by repeated-measures ANOVA. We found significant differences in waveform amplitude between deviant and standard stimuli (*F*_1_,_10_ = 21.2892, *p* = 0.0010). In addition to the main effects of stimulus type, there was a significant effect of call type (*F*_1_,_10_ = 33.4938, *p* = 0.0002). We did not find significant differences in ERPs between channels (*F*_1_,_12_ = 1.5952, *p* = 0.2352).

The significant differences between deviant and standard ERPs, both to pure tones and natural vocalizations, were observed in the negative component ranging from 165 to 270 ms after stimulus onset. This is comparable with MMR in human and other species.

### Possible Cause of the Negative Shift of Event-Related Potentials to Deviant Stimuli Is Stimulus-Specific Adaptation

In the oddball sequence, the probability differed between the standard and deviant stimuli. Repeated exposure to a sound causes SSA and decreased neural responses to repeated playback of a sound (Chew et al., 1995; Stripling et al., 1997; Kozlov and Gentner, 2014). To examine the effect of the probability, we compared responses to the deviant stimulus in the oddball paradigm and the control stimulus in the randomized standard sequence (in both cases, it is the same physical stimulus) (Figure 2D). If MMR in the oddball sequence reflects sensory memory-based responses, MMR will be detected using the randomized standard sequence as a control. In contrast, if SSA is the principal cause of the significant difference in response to the deviant and standard stimuli in the oddball sequence, MMR will not be detected using the randomized standard sequence. The mean amplitude of the ERPs at 165–190 ms was analyzed by repeated-measures ANOVA. No significant differences in ERPs were observed in stimulus type or channels (stimulus type: *F*_1_,_12_ = 1.9127, *p* = 0.1919; channel: *F*_1_,_12_ = 0.0763, *p* = 0.7871). ERPs were significantly different between frequencies of sound (*F*_3_,_36_ = 11.4555, *p* < 0.0001). Furthermore, in addition to equally probable stimuli, we compared responses to predictable tones in the regular pattern sequence and unpredictable deviant tones in the oddball sequence to examine the effect of predictability (Figure 2E). If MMR reflects prediction error elicited by the deviant, MMR will be detected using the regular pattern sequence as a control. The mean amplitude of the ERPs at 165–190 ms was analyzed by repeated-measures ANOVA. There were no significant differences in ERPs between stimulus type or channels (stimulus type: *F*_1_,_12_ = 1.9291, *p* = 0.1901; channel: *F*_1_,_12_ = 0.2818, *p* = 0.6052). ERPs were significantly different between frequencies of sound (*F*_1_,_12_ = 6.6616, *p* = 0.0241).

Considering that there were significant differences in the ERPs between the deviant and standard stimuli in the oddball sequence but no significant differences between the deviant stimulus in the oddball sequence and control stimuli in the randomized standard sequence, these results indicate that stimulus probability affected the waveform of ERPs. High stimulus probability reduced the amplitude of later negative peaks of ERPs. In contrast, the predictability of the stimuli did not affect ERP amplitude, as no mismatch response was seen when regular pattern sequences were presented as a control. However, another possibility for this result is that the rules of regular pattern sequence were not detected in NCM, and ERPs did not properly reflect stimulus predictability.

### The Event-Related Potentials in Caudomedial Nidopallium Were Sensitive to Differences in Triplet Temporal Pattern

**FIGURE 3:**
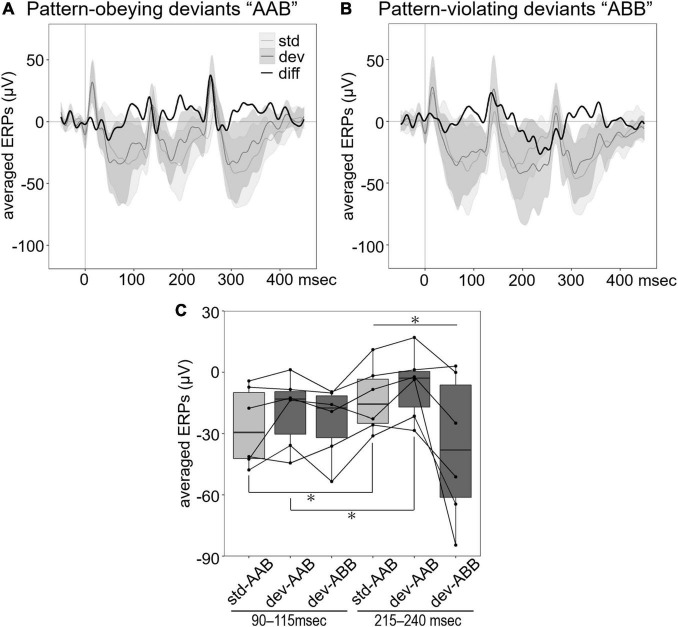
Averaged ERPs to triplet sequences. **(A)** Group averaged ERPs to standard “AAB” (light gray line) and pattern-obeying deviants “AAB” (dark gray line). **(B)** Group averaged ERPs to standard “AAB” (light gray line) and pattern-violating deviants “ABB” (dark gray line). Black line in both **(A,B)** shows the difference wave obtained by subtracting the ERPs to standard stimuli from the ERPs to deviant stimuli. The shading indicates the range of 95% CI of the waves. **(C)** Each box shows the quartiles of the ERPs to standard (light gray) and deviant (dark gray) stimuli in each time window (90–115 ms for the first sound, 215–240 ms for the second sound in the triplet sequence). Each dot represents the averaged ERPs for each individual (*n* = 6). The line connects data of the same individual. The negative waveform of ERPs to the second sound of triplet sequences reduced in standard “AAB” and pattern-obeying deviants “AAB” compared with pattern-violating deviants “ABB” (repeated measures ANOVA, *p < 0.05).

To further examine sensitivity to the sequence order of sound patterns, we presented laboratory-created triplet sequences using natural calls. To evaluate the differences between ERPs to standard “AAB” and pattern-obeying deviants “AAB” (Figure 3A) or pattern-violating deviants “ABB” (Figure 3B), we focused on first and second negative components and compared with an averaged amplitude of LFP for each time window: 95–115 ms and 215–240 ms after stimulus onset (Figure 3C). These windows correspond to 95–115 ms after stimulus onset of the first and second sounds in the triplet sequence. Since the change in the pattern occurs in the second sound of the pattern-violating deviants, ERPs to second sounds are analyzed to investigate MMR and compared with the preceding ERPs to the first sound, which reflected most probable acoustical differences. Pattern-obeying deviants consisted of two different calls that physically differed from the standards but had the same pattern. Pattern-violating deviant differed from the standard both physically and in the pattern. The mean amplitude of the ERPs at each time window was analyzed by repeated-measures ANOVA. There was a significant effect of stimulus pattern (*F*_2_,_20_ = 4.6968, *p* = 0.0213) and an interaction between stimulus pattern and time window (*F*_2_,_20_ = 5.1440, *p* = 0.0158). The simple effect of the interaction showed that ERPs at 215–240 ms, corresponding to the negative peak, differed among stimulus patterns (*F*_2_,_20_ = 5.4861, *p* = 0.0126). Furthermore, the mean amplitude of the ERPs to standard “AAB” (*F*_1_,_10_ = 7.5885, *p* = 0.0203) and pattern-obeying deviants “AAB” (*F*_1_,_10_ = 7.4773, *p* = 0.0210) were significantly different between time windows, but those of pattern-violating deviants “ABB” were not (*F*_1_,_10_ = 2.1542, *p* = 0.1729). These results suggest that standard “AAB” and pattern-obeying deviants “AAB” showed a decreased negative shift of the ERPs to the second call in the triplet sequence compared with the first call, but the pattern-violating deviants “ABB” did not. This suggests a fundamental difference between the “AAB” deviants (standard and pattern-obeying) and the pattern-violating deviants “ABB.”

## Discussion

In this study, we demonstrated MMR in the NCM of Java sparrows in response to deviant pure tones and natural vocalizations in an oddball paradigm in which differences in the physical characteristics of the stimuli were controlled. However, in comparison, significant mismatch responses were not observed in the ERPs to the sequences controlled for probability and predictability of stimuli. These results indicate that the probability of stimulus frequency affected the shape of ERP waveforms. Furthermore, to investigate the sensitivity to the pattern of sequence, we compared the ERPs with three types of triplet sound sequences. The ERPs to pattern-violating deviants “ABB” differed significantly from ERPs to “AAB” triplet sequences. This suggests that NCM has the sensitivity to differences in the elemental order.

In the oddball sequence, MMR appeared at 165–270 ms after the sound onset. This is consistent with previous studies in humans, primates, dogs, cats, rodents, and pigeons, in which a significant decrease in ERP amplitude is seen in response to deviant stimuli. The peak of human MMN usually appears at approximately 150–250 ms from the onset of stimuli (Näätänen et al., 2007). Non-human animal MMR sometimes exhibits shorter latencies than human MMN (Shiramatsu and Takahashi, 2021): 48–150 ms in macaque (Javitt et al., 1992; Javit et al., 1994; Molholm et al., 2005; Gil-Da-Costa et al., 2013), 37–131 ms in marmosets (Komatsu et al., 2015), 160–200 ms in dogs (Howell et al., 2011, 2012), 30–74 ms in cats (Csépe et al., 1987; Pincze et al., 2001), 50–150 ms in rodents (Umbricht et al., 2005; Ehrlichman et al., 2008; Shiramatsu et al., 2013; Christianson et al., 2014; Harms et al., 2014), and 50–250 ms in pigeons (Schall et al., 2015). The differences in MMR latency might result from differences in the network, structure, and size of the brain, although we cannot rule out the possibility that differences are due to the neural recording technique, experimental conditions (e.g., under anesthesia or freely behaving), or position of electrodes.

To separate MMN, reflecting a genuine deviance detecting property, from SSA, we performed recordings in which a randomized standard sequence and a regular pattern sequence were presented. Our results showed that using these two types of sequences in the oddball task did not elicit significant levels of deviance detection. At present, several studies have presented randomized standard sequences and successfully recorded MMN in human (Schröger and Wolff, 1996) and MMN-like responses in rats (Nakamura, 2011; Shiramatsu et al., 2013; Harms et al., 2014) and in pigeons (Schall et al., 2015), while some studies using macaques failed (Fishman and Steinschneider, 2012; Kaliukhovich and Vogels, 2014). Regarding the regular pattern sequence, some studies on human (Ruhnau et al., 2012) and rodents (Parras et al., 2017) demonstrated genuine deviance-detecting responses. However, Harms et al. could not find evidence of SSA-independent responses in the rat auditory cortex using this paradigm (Harms et al., 2014). Since the advantages and disadvantages of the control sequences are still controversial, further studies and considerations are needed for characterizing genuine deviance detection.

We used natural vocalizations, song elements, and calls of Java sparrows as stimuli for the oddball sequence and triplet sequence paradigm. Detection of deviants in natural auditory scenes is an important component of determining which ecological events are relevant to behavior and require an attention switch for further processing (Näätänen et al., 2001). This suggests that detecting deviance in natural complex sounds is critical for animal survival. Electrophysiological recording studies have reported that the auditory forebrain of songbirds shows larger responses to deviant calls than to standard calls using an oddball paradigm (Beckers and Gahr, 2012; Dong and Vicario, 2018). We demonstrated MMR elicited by deviant sounds using an oddball paradigm with song elements and calls. Song elements and calls for stimuli were sampled from a single and two individuals, which seemed to be a small sample size to generalize the result. However, MMR is also elicited by the changes in more complex triplet temporal patterns using the calls from five individuals. These suggest that MMRs reflect the deviance detection of natural communicative sounds. Song elements and calls elicited negative peaks that were larger in amplitude and longer in duration compared with those elicited by pure tones. In addition to containing more acoustic information than pure tones, call and song elements might attract more attention, which can modulate ERPs (Picton et al., 1971; Hromádka and Zador, 2007).

Previous studies indicated that human MMN can be elicited by categorical changes and violation of abstract rules of sequences (Tervaniemi et al., 2001; Pulvermu and Shtyrov, 2003). The detection of categorical changes is considered to reflect higher cognitive function rather than the effects of adaptation. Some studies using primates and rats demonstrated MMR elicited by the change in melodic contours (Ruusuvirta et al., 2007) and grammar-like sequences (Astikainen et al., 2014; Attaheri et al., 2015). In the triplet sequence paradigm, we detected MMR at 95–115 ms after the second sound onset (215–240 ms after the stimulus onset) specifically in pattern-violating sequences “ABB.” Our result is consistent with a previous report of sequence sensitivity in NCM neurons using a triplet sequence oddball task (Ono et al., 2016), and it supports that MMR in this paradigm, such as MMN in humans, reflects genuine deviance detection rather than adaptation.

The current experiments showed that MMR elicited by oddball paradigm using pure tone could arise from SSA, but MMR elicited by violation of abstract rules of the sequences primarily reflect deviance detection in NCM. Songbirds including Java sparrows learn and produce songs composed of complex temporal sequences. They communicate by call interactions and maintain pair-bond and group. The acoustic structure and temporal pattern of successive calls reflect social relationships (Elie et al., 2011). Thus, they are sensitive to sound sequences. Even though more experiments have to be conducted, we believe our study shows that MMRs in Java sparrows share some characteristics with MMN and may provide insights into the electrophysiological properties of MMR and SSA, which represent sound sequence processing.

## Conclusion

We detected MMR in the songbird NCM to deviants with an oddball paradigm, whereas significant negative shifts were not detected using randomized standard sequences and regular pattern sequences. These results indicate that MMR demonstrated in the oddball paradigm reflects the adaptation to a repeated standard sound. Using a triplet sequence paradigm, we successfully detected MMR elicited by violation of the pattern of sound sequence, which is considered to reflect the ability to extract structural information. Our findings can be used as a basis for understanding the neural mechanisms and functional role of MMR in songbirds and for comparing MMR with MMN in humans. However, it remains necessary to optimize the paradigm to investigate how neural adaptation and genuine deviance detecting properties contribute to ERP waveforms and further to combine recordings with pharmacological manipulations (e.g., antagonists of NMDA).

## Data Availability Statement

The raw data supporting the conclusions of this article will be made available by the authors, without undue reservation.

## Ethics Statement

The animal study was reviewed and approved by the Institutional Animal Care and Use Committee at the University of Tokyo.

## Author Contributions

CM and KO designed this study and wrote the manuscript. CM performed the experiments and data analysis. Both authors contributed to the article and approved the submitted version.

## Conflict of Interest

The authors declare that the research was conducted in the absence of any commercial or financial relationships that could be construed as a potential conflict of interest.

## Publisher’s Note

All claims expressed in this article are solely those of the authors and do not necessarily represent those of their affiliated organizations, or those of the publisher, the editors and the reviewers. Any product that may be evaluated in this article, or claim that may be made by its manufacturer, is not guaranteed or endorsed by the publisher.
